# Philosophy in Sport Made Science in Earnest: Being an Attempt to Illustrate the First Principles of Natural Philosophy by the Aid of the Popular Toys of Youth

**Published:** 1847-05

**Authors:** 


Art. VI.—*Philosophy in Sport Made Science in Earnest;* being an attempt to Illustrate the First Principles of Natural Philosophy by the aid of the Popular Toys of Youth. From the sixth and greatly improved London edition. 12mo. pp. 432. Philadelphia: Lea and Blanchard: 1847.

The title of this little book is attractive, and its external appearance is fully as much so; the publishers have sent it forth in a beautiful dress. Philosophy ought to be made pleasing to youth, for in itself we hold with Milton, that it is "not harsh and crabbed as dull fools suppose." The mind naturally craves knowledge, and the youthful mind is especially inquisitive and active. It must therefore be the fault of the systems in vogue, that learning is felt to be an oppressive task, instead of an ever recurring, exhaustless pleasure. How far the author of this enterprise has succeeded in making philosophy a pastime for youth, we are not able to say from any thorough examination of his book, but it is much in its favor that it has passed through so many editions. Any scheme which promises an alleviation of human suffering, such as that endured by children at school, we hold to be worthy the consideration of every philanthropist, and such is the aim of the anonymous little volume before us.

